# Pharmacogenomics in the United Arab Emirates: advances in population genomics and challenges in clinical implementation

**DOI:** 10.3389/fphar.2026.1902530

**Published:** 2026-09-07

**Authors:** Abderraouf Haitamar, Shahid Eiyad Abu-Nada, Mariam Wed Eladham, Bushra Mdkhana, Narjes Saheb Sharif-Askari, Rabih Halwani, Fatemeh Saheb Sharif-Askari

**Affiliations:** 1 Research Institute for Medical and Health Science, University of Sharjah, Sharjah, United Arab Emirates; 2 Department of Pharmacy Practice and Pharmacotherapeutics, College of Pharmacy, University of Sharjah, Sharjah, United Arab Emirates; 3 Department of Clinical Sciences, College of Medicine, University of Sharjah, Sharjah, United Arab Emirates; 4 Prince Fahad Bin Sultan chair for Biomedical Research, University of Tabuk, Tabuk, Saudi Arabia; 5 College of Medicine, Alfaisal University, Riyadh, Saudi Arabia; 6 Health and Wellbeing, NEOM, Tabuk, Saudi Arabia; 7 King Saud University Celiac Disease Research Chair, department of Pediatric, College of Medicine, King Saud University, Riyadh, Saudi Arabia

**Keywords:** clinical implementation, emirati genome program, pharmacogenomics, precision medicine, United Arab Emirates

## Abstract

Pharmacogenomics (PGx) is a key component of precision medicine, linking genetic variation to drug response, effectiveness, and toxicity. However, Middle Eastern and North African (MENA) populations remain underrepresented in global genomic databases, limiting PGx’s clinical utility in these regions. This review examines the current state of pharmacogenomics in the United Arab Emirates (UAE), focusing on genomic initiatives, population-specific variation, and challenges in clinical implementation. National genomic programs, including the Emirati Genome Program, have begun to address this gap by generating ancestry-specific data. Studies of UAE populations reveal clinically relevant variation in pharmacogenes that affect commonly used drugs such as warfarin, clopidogrel, and statins, as well as rare and understudied variants with potential functional impact. Additional evidence highlights the role of HLA diversity and genetic factors in metabolic and inflammatory conditions. Despite these advances, implementation remains constrained by gaps in healthcare professional education, infrastructure, regulatory frameworks, and public awareness. The UAE is well-positioned to advance PGx through targeted clinical integration, the development of local reference systems, and strengthened policy support. These efforts are essential to enable effective, population-specific precision medicine in the region.

## 1 Introduction

The completion of the Human Genome Project in 2003 marked a transformative milestone in predictive and personalized medicine (International Human Genome Sequencing, 2004; Collins et al., 2003; Lander et al., 2001), enabling the emergence of Pharmacogenomics (PGx) as a central pillar of precision medicine by linking interindividual genetic variation to differences in drug effectiveness, toxicity, and optimal dosing (Pirmohamed, 2023).

Globally, several population-based genomic programs have been established to characterize human genome diversity and support the implementation of precision medicine. However, populations from the Middle East and North Africa (MENA) region remain underrepresented in major public genomic resources. For example, gnomAD and TOPMed contain substantially larger numbers of individuals of European ancestry than individuals of Middle Eastern or Arab ancestry, limiting the availability of population-specific allele-frequency data for the region (Karczewski et al., 2020; Taliun et al., 2021; Ateia et al., 2023). This underrepresentation may result in variants that are relatively common in Arab populations being incorrectly classified as rare or of uncertain significance. It may also reduce the accuracy of disease-gene discovery, variant interpretation, polygenic risk prediction, and pharmacogenomic recommendations (Karczewski et al., 2020; Taliun et al., 2021; Ateia et al., 2023). Consequently, many genomic and pharmacogenomic analyses continue to rely on reference datasets that may not adequately reflect the genetic architecture of populations in the UAE and the wider MENA region. To address this gap, national genomic initiatives have been established in the UAE, including projects designed to characterize Emirati genomic diversity and generate population-specific reference data (Al-Ali et al., 2018; AlSafar et al., 2019).

Recognizing these limitations, the country has emerged as one of the regional leaders in advancing genomic medicine initiatives. Driven by the country’s vision for innovation and healthcare modernization, the UAE has launched several large-scale genome programs aimed at building population-specific genomic resources and supporting the integration of precision medicine into clinical practice. These efforts are practically important given the high rates of consanguinity and the growing burden of chronic diseases within the region, both of which increase the relevance of genomics-guided healthcare strategies (Ateia et al., 2023).

This review summarizes the current landscape of pharmacogenomics (PGx) in the UAE, highlighting advances in population genomics, emerging evidence for clinically actionable pharmacogenetic variation, healthcare professional and public readiness, and the major scientific, educational, economic, and regulatory challenges that must be addressed to facilitate the integration of precision medicine into routine clinical care. The overall framework and key components of pharmacogenomics implementation in the UAE are summarized in Figure 1.

**FIGURE 1:**
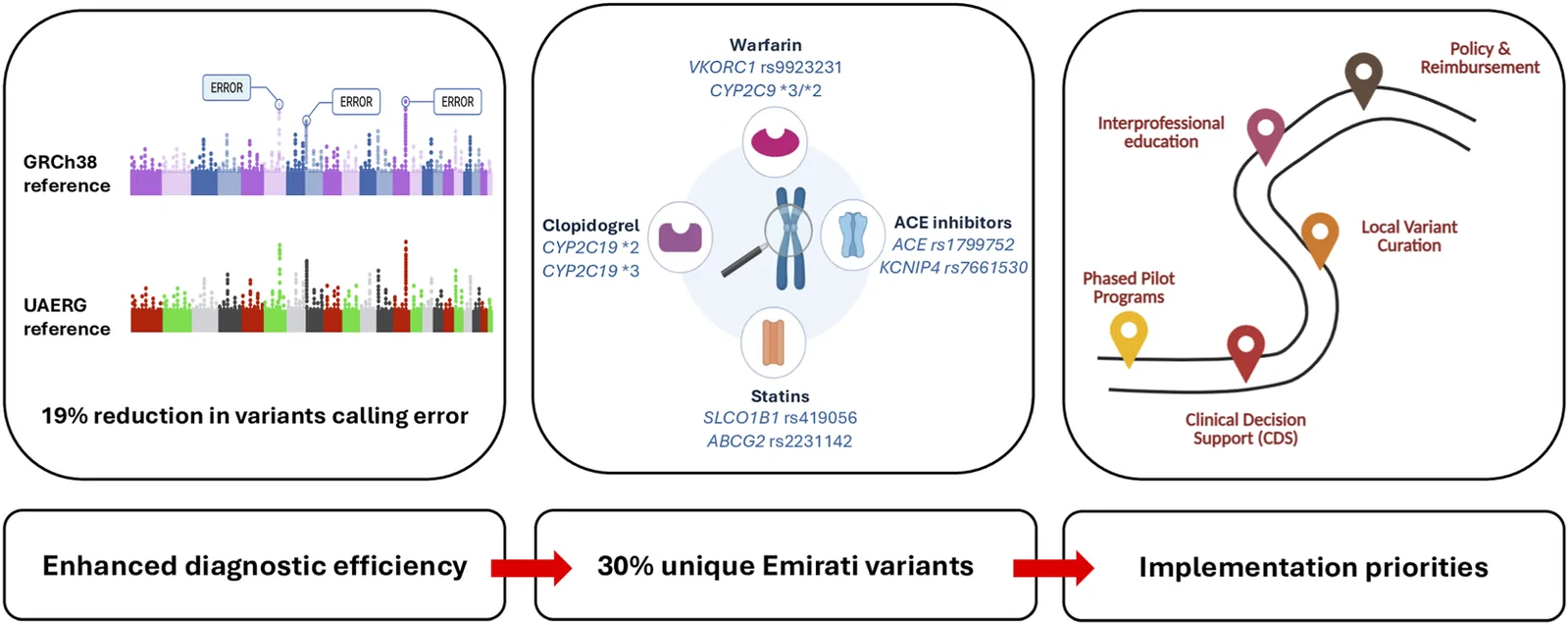
Pharmacogenomics implementation framework in the United Arab Emirates. The framework highlights (1) enhanced diagnostic accuracy using the UAE Reference Genome (UAERG), with a 19% reduction in variant calling errors compared with the GRCh38 reference; (2) identification of ∼30% unique Emirati pharmacogenomic variants across key drug classes, including antiplatelet agents, anticoagulants, antihypertensives, and statins; (3) prioritized implementation enablers spanning clinical decision support, phased pilot programs, variant curation, interprofessional education, and supportive policy and reimbursement. Abbreviations: ACE, angiotensin-converting enzyme; CDS, clinical decision support; UAERG, United Arab Emirates Reference Genome.

## 2 Literature search methodology

The literature search was conducted in April 2026 using PubMed/MEDLINE, Scopus, and Web of Science. Search terms included “United Arab Emirates”, “UAE”, “Emirati”, “pharmacogenomics”, “pharmacogenetics”, “drug response”, and “precision medicine”, with equivalent search strategies adapted to each database. Only English-language, peer-reviewed publications were included. Duplicate records, conference abstracts, editorials, non-English publications, and studies outside the scope of this review were excluded. Additional relevant articles were identified through manual screening of the reference lists of eligible publications.

## 3 Genomic foundations in the UAE

A strong pharmacogenomics framework depends on comprehensive population-specific genomic knowledge, and the UAE has begun establishing this foundation through national and institutional initiatives. The Emirati Genome Program was developed to generate large-scale genomic data representative of the Emirati population and to address the historical lack of Middle Eastern representation in international genomic repositories (Fortina et al., 2014). This initiative is particularly important because PGx-guided therapy, disease risk prediction, and variant interpretation are more accurate when based on genomic reference datasets that reflect the genetic background of the local population (Abbas et al., 2025a; Marzouka et al., 2024).

Research conducted within the country has already demonstrated the value of developing local genomic references. Early whole-genome sequencing studies of Emirati nationals revealed the unique ancestry composition of the population, including a substantial Central and South Asian ancestry composition (51%), reflecting the UAE’s historical trade routes and demographic diversity (AlSafar et al., 2019). Building upon these findings, the United Arab Emirates Reference Genome (UAERG) was developed using high-coverage genomic sequencing data from 153 Emirati individuals. The UAERG significantly improved variant detection accuracy and reduced variant-calling errors by 19% compared with standard international reference genomes (GRCh38), thereby enhancing the reliability of local genomic analysis (Daw Elbait, 2021). These advances are particularly relevant to PGx because the accurate identification of actionable pharmacogenomic variants depends on reference systems that adequately represent the allele frequencies and genetic diversity of the target population (Daw Elbait, 2021).

Importantly, studies conducted in Emirati cohorts have demonstrated that many clinically relevant pharmacogenetic variants are either rare or unique to the UAE population and may not be adequately represented in commonly used international PGx databases. Variants affecting key pharmacogenes, including *CYP2C19* and other genes involved in cardiovascular and metabolic drug responses, have shown population-specific distributions among Emiratis (Khasawneh et al., 2024). Consequently, reliance solely on globally established star alleles and European-centric PGx interpretation frameworks may overlook clinically important variants relevant to local patients. This highlights the need for UAE-specific genomic databases, locally curated PGx interpretation systems, and regionally validated prescribing guidelines to ensure safe and effective implementation of precision medicine (Marzouka et al., 2024).

In parallel with national initiatives, the UAE has also actively participated in regional collaborations aimed at advancing genomic medicine across the Arab world. Conferences and collaborative initiatives such as the Pan Arab Human Genetics Conferences have played an important role in promoting genomic research, fostering scientific collaboration, and supporting the translation of genomics into public health and clinical applications within MENA populations (Fortina et al., 2014; Jamalalail et al., 2026).

## 4 Pharmacogenomic evidence in clinical practice

### 4.1 Cardiovascular pharmacogenomics

Cardiovascular medicine currently represents the leading area of pharmacogenomic implementation in the UAE. The major pharmacogenomic findings reported across different therapeutic areas in UAE populations are summarized in Table 1. The first pilot implementation study demonstrated the feasibility and potential clinical utility of integrating pharmacogenomic testing into routine cardiovascular care (Al-Mahayri et al., 2022). Among 160 patients receiving clopidogrel, warfarin, atorvastatin, or rosuvastatin, 96% received at least one pharmacogenomic-guided therapeutic recommendation, reflecting the high prevalence of actionable pharmacogenomic variants in this population (Al-Mahayri et al., 2022). Given the substantial burden of cardiovascular disease in the UAE, these findings highlight the potential of pre-emptive pharmacogenomic testing to optimize drug selection and dosing, reduce adverse drug reactions, improve treatment adherence, and ultimately enhance long-term clinical outcomes (Abbas et al., 2025a; Al-Mahayri et al., 2022).

**TABLE 1:** Summary of clinically relevant pharmacogenomic evidence reported in UAE populations.

Disease/Clinical Area	Associated Drugs	Genes or Genetic Markers	Key Findings	Clinical Significance	Population Subgroups Affected	References
Cardiology	Clopidogrel	*CYP2C19, ABCB1, PON1, P2Y12*	High prevalence of variants reduces drug activation, resulting in intermediate or poor metabolizers.	Increased risk of treatment failure, recurrent ischemic events, or stent thrombosis.	Emirati nationals	Khasawneh et al. (2024)
	Warfarin	*VKORC1*	VKORC1 variants are significant predictors of dose requirements; models including age and markers explain substantial dose variability.	Narrow therapeutic index means inaccurate dosing risks serious bleeding or thrombotic complications.	Emirati nationals	Al-Mahayri et al. (2019)
	Cardiovascular drugs	Rare missense *CYP2C19* variants	Discovery of rare missense variants that reduce enzyme activity, which are missed by standard European/East Asian panels.	Standard clinical genotyping panels may underperform in understudied ancestries.	Emirati individuals	Saleous et al. (2025)
	Rosuvastatin, Atorvastatin	*ABCG2, SLCO1B1*	Variants associated with drug discontinuation, liver enzyme elevations, and muscle-related adverse effects.	Statin intolerance leads to poor adherence and suboptimal lipid control.	Multiethnic UAE population	Alqasrawi M. N. N. et al. (2025), Alqasrawi M. N. et al. (2025)
Cardiology/Hypertension	Angiotensin-converting enzyme (ACE) inhibitors	*ACE* rs1799752, *KCNIP4* rs7661530	Genetic associations found with ACE inhibitor–induced cough in hypertensive patients.	Helps anticipate which patients are likely to discontinue therapy due to adverse effects.	Multiethnic UAE cohort	Altoum et al. (2025)
Metabolic/Obstetrics	Sulphonyureas	Monogenic diabetes genes (*HNF1A, ABCC8, KCNJ11*)	Pathogenic variants identified in women with gestational diabetes linked to persistent dysglycemia.	Higher risk of non-autoimmune diabetes within 5 years after pregnancy; implies need for postpartum surveillance.	Emirati women	Daggag et al. (2022)
	Insulin	Monogenic diabetes genes (*INS Variant Carriers*)				
Metabolic/Surgery	Metformin and GLP-1 Agonists	Host genetic variation and microbial factors (rs11830243 and rs6978118)	Biological response to bariatric surgery (inflammatory biomarkers and metabolites) is genetically mediated.	Supports integrated model of personalized therapeutic response in metabolic disease.	Emirati patients	Almazrouei et al. (2025)
	Antihypertensive medications	Host genetic variation and microbial factors (rs9490306, rs62207434, and rs34606058)				
Immunogenetics	Sulfamehoxazole/Trimethoprim	HLA-C*06:02	Second most prevalent allele in the cohort at 14.04%	Associated with hypersensitivity reactions	UAE Citizens	Marzouka et al. (2024)
	Aspirin	HLA-DPB1*03:01	Third most prevalent allele in the cohort at 8.73%	Linked to aspirin sensitivity		
	Allopurinol	HLA-B*58:01	Detected at a frequency of 4.2%	Linked to allopurinol sensitivity		
	Carbamazepine	HLA-A*31:01	Exhibits a frequency of 2.9%	Linked to carbamazepine sensitivity		
	Abacavir and Flucloxacillin	HLA-B*57:01	Observed at a frequency of 0.53%	Linked to Antiviral and Antibacterial drug sensitivity		
	Phenytoin, Lamotrigine, Oxcarbamazepine, and Antiepileptics	HLA-B*15:02	Observed at the lowest frequency of 0.44%	Linked to mutliple Antiepileptic drug sensitivity		

The table summarizes the available pharmacogenomic studies conducted in the UAE, populations across therapeutic areas. It highlights the drugs investigated, associated genetic variants, key findings, clinical implications, and study populations.

Abbreviations: HLA, human leukocyte antigen; UAE, united arab emirates.

Warfarin represents one of the clearest examples of genotype-guided therapy in the UAE. *VKORC1* variants, particularly rs9923231 and rs61742245, together with patient age, explained approximately half (50.7%) of the variation in warfarin dose requirements among patients (adjusted R^2^ = 0.507; P < 0.05). These findings demonstrate that genetic variation and age are major determinants of the dose required to achieve effective and safe anticoagulation, supporting the implementation of genotype-guided warfarin dosing in the UAE (Al-Mahayri et al., 2019). This finding is clinically important because warfarin has a narrow therapeutic index, and inaccurate dosing can result in serious bleeding or thrombotic complications (Estimation of the Warfarin Dose, 2009). Supporting the clinical relevance of these findings, a pilot pharmacogenomic implementation study involving 160 cardiovascular patients in the UAE reported that 80% of patients carried actionable *VKORC1* or *CYP2C9* variants that could be incorporated into genotype-guided warfarin dosing algorithms, highlighting the feasibility and potential clinical benefit of implementing pharmacogenomic-guided anticoagulation in routine practice (Al-Mahayri et al., 2022).

Clopidogrel response is another important pharmacogenomic consideration in the Emirati population. Whole-exome sequencing of 298 healthy Emirati individuals identified a high prevalence of clinically relevant *CYP2C19* variants associated with reduced clopidogrel activation. Overall, approximately 40% of the cohort were predicted to have intermediate or poor metabolizer phenotypes, suggesting that a substantial proportion of Emirati patients may benefit from alternative antiplatelet agents, such as prasugrel or ticagrelor, rather than clopidogrel (Khasawneh et al., 2024). In addition, variants in *ABCB1*, *PON1*, and *P2Y12* were highly prevalent, with more than half of the cohort carrying variants that may further influence clopidogrel transport, bioactivation, and platelet response (Khasawneh et al., 2024). These observations were further supported by a pilot pharmacogenomic implementation study in 160 cardiovascular patients, in which 46.9% of patients received a recommendation to avoid clopidogrel or consider an alternative antiplatelet agent based on their *CYP2C19* genotype (Al-Mahayri et al., 2022).

Statin pharmacogenomics has also been extensively studied in UAE populations. Recent prospective studies have shown that genetic variation in *SLCO1B1* and *ABCG2* significantly affects statin tolerability, safety, and treatment adherence (Alqasrawi et al., 2024; Alqasrawi M. N. N. et al., 2025; Alqasrawi M. N. et al., 2025). Population-based whole-exome sequencing of 242 Emirati individuals demonstrated a 29.8% and 5.4% of individuals predicted to have decreased and poor *SLCO1B1* function, respectively, indicating an increased susceptibility to statin-associated adverse effects (Alqasrawi et al., 2024). In a prospective multicenter cohort study, 422 adults prescribed rosuvastatin were followed for 12 months. Patients carrying the *ABCG2* rs2231142 T/T genotype had a 4.4-fold higher risk of treatment discontinuation than non-carriers (HR = 4.40, P < 0.001), followed by those with the *ABCG2* G/T genotype (HR = 1.75), primarily because of treatment-related adverse effects, highlighting the potential value of *ABCG2* genotyping to improve long-term treatment adherence (Alqasrawi M. N. N. et al., 2025). In a sub-analysis of the prospective EmHeart Study involving 675 patients receiving rosuvastatin or atorvastatin, rosuvastatin users carrying the *ABCG2* rs2231142 variant had an approximately threefold increased risk of liver enzyme elevation, particularly among East Asian patients (P < 0.005). In contrast, atorvastatin users carrying the *SLCO1B1* rs4149056 variant exhibited a twofold increased risk of statin-associated muscle symptoms (SAMS), with higher rates observed among females and individuals of Arab ethnicity (P < 0.05) (Alqasrawi M. N. et al., 2025). These findings are further supported by the first pharmacogenomic implementation study in cardiovascular patients in the UAE, which demonstrated that 31.5% of patients carried at least one actionable *SLCO1B1* rs4149056 allele associated with an increased risk of statin-induced myopathy (Al-Mahayri et al., 2022).

Another notable pharmacogenomic finding in the UAE is the identification of genetic predictors of angiotensin-converting enzyme inhibitor (ACEI)-induced cough. In a multicentre study of 107 hypertensive patients receiving ACEIs, the *ACE* rs1799752 I/D genotype (adjusted P = 0.046) and the *KCNIP4* rs7661530 T/T genotype (adjusted P = 0.025) were significantly associated with an increased risk of ACEI-induced cough. Furthermore, patients who developed cough had significantly lower ACE plasma levels than those without cough (P = 0.0014), with the greatest difference observed among individuals carrying the *ACE* rs1799752 I/D genotype (P = 0.0061), providing biological support for the observed genetic associations. Although these findings require validation in larger cohorts, they represent the first evidence from the UAE and the Middle East linking genetic variation and *ACE* plasma levels to ACEI-induced cough, highlighting the potential of pharmacogenomics to identify patients at increased risk of treatment intolerance and guide personalized antihypertensive therapy (Altoum et al., 2025).

### 4.2 Rare variants and functional relevance

One of the most important contributions of UAE-based pharmacogenomics research is the recognition that rare and understudied variants may be clinically significant. In the case of *CYP2C19*, investigations identified several rare missense variants that reduced enzyme activity and would not have been captured adequately through testing strategies focused only on the most common alleles (Shubbar, 2024). This is a major finding for PGx implementation because many current clinical genotyping panels are optimized around frequently studied variants from European or East Asian populations and may therefore perform suboptimally in understudied populations (Saleous et al., 2025).

These data reinforce the concept that pharmacogenomics should not be limited to a small set of globally common variants. Functional characterization of rare local alleles through computational prediction and experimental validation is necessary to determine whether these variants are clinically actionable and should be incorporated into future testing panels. Although several rare *CYP2C19* variants identified have demonstrated altered enzymatic activity (Saleous et al., 2025), their clinical significance requires validation in larger prospective studies before routine incorporation into prescribing guidelines. Continued population-specific discovery research will therefore remain essential as pharmacogenomics implementation advances within the UAE healthcare system (Saleous et al., 2025).

### 4.3 Metabolic and obstetric contexts

Although cardiovascular PGx currently dominates the UAE literature, emerging evidence from metabolic and obstetric studies is expanding the clinical scope of precision medicine in the country. In a cohort of 370 Emirati women with gestational diabetes, seven women (2%) were identified as carriers of pathogenic variants in monogenic diabetes genes, including *GCK*, *HNF1A*, *INS*, *ABCC8*, and *KCNJ11* (Daggag et al., 2022). Carriers were significantly more likely to develop postpartum hyperglycemia or type 2 diabetes than non-carriers, with a 1.8-fold increased risk of dysglycemia (95% CI: 1.1–2.9; P = 0.02) and a 2.5-fold increased risk of type 2 diabetes (95% CI: 1.3–4.8; P = 0.009) during follow-up (Daggag et al., 2022). Although pathogenic variants were identified in only a small proportion of women, these findings have important implications for postpartum risk stratification and individualized long-term management by identifying a subgroup of patients at substantially increased risk of persistent hyperglycemia and type 2 diabetes after pregnancy (Daggag et al., 2022).

Additional work in Emirati patients undergoing bariatric surgery showed that improvements in inflammatory biomarkers, metabolite profiles, and gut microbiome composition were influenced by interactions between microbial factors and host genetic variation (Almazrouei et al., 2025). While this does not represent classical drug-gene pharmacogenomics in the narrowest sense, it strongly supports the broader precision medicine principle that individual biological response is partly genetically mediated. These findings help expand precision medicine concepts beyond drug metabolism alone to a more integrated model of personalized therapeutic response in metabolic disease (Almazrouei et al., 2025).

### 4.4 HLA diversity and precision medicine

The UAE population also exhibits a distinct HLA allele profile shaped by regional ancestry patterns and consanguinity, where consanguinity rates range from 39% to 54%, compared with fewer than 5% in many Western populations (Hamamy, 2012; al-Gazali et al., 1997). Studies have identified 132 novel HLA alleles linked to disease susceptibility and drug-associated toxicity, including 32 rare alleles with allele frequencies below 0.003 that were completely absent from 99 global populations studied (Marzouka et al., 2024; Arnaiz-Villena et al., 2019). This is highly relevant to pharmacogenomics because HLA variation is already known internationally to influence serious adverse drug reactions for several medicines. A population-specific HLA reference framework could therefore improve not only transplant compatibility but also medication safety through more locally appropriate risk prediction (Marzouka et al., 2024).

The significance of HLA-related findings lies in their contribution to a broader precision medicine ecosystem. PGx implementation is often discussed in terms of cytochrome P450 enzymes and transporter proteins, but the UAE evidence shows that immune-related genetic architecture must also be considered in the design of future national testing strategies. This supports a more comprehensive view of clinically actionable genomics, one that includes metabolism, transport, immune toxicity, and disease predisposition as interconnected components of precision care (Marzouka et al., 2024).

## 5 Patient awareness and public attitudes

Successful pharmacogenomics implementation depends not only on laboratory capability but also on public understanding and social acceptance. Many studies indicate that although general genomic literacy remains limited, with only 7% of the public in the UAE demonstrating good knowledge of genomics, willingness to participate in research and to undergo genetic testing was relatively high, with 76.9% of the public agreeing particularly when individuals perceive a family history of disease or a clear health benefit. This mismatch between positive attitudes and limited knowledge is important because it suggests that low awareness is not necessarily a sign of resistance, but rather a remediable educational gap (Daw Elbait, 2021; Rahma et al., 2023).

At the same time, the available evidence shows that concerns about privacy, confidentiality, misuse of genetic information, and potential insurance consequences remain substantial. These concerns can weaken trust and reduce long-term engagement if policy frameworks are not sufficiently transparent and protective. In culturally diverse populations, sensitive and accessible public communication is therefore necessary to ensure that enthusiasm for genomic medicine is translated into informed participation rather than passive acceptance (Khasawneh et al., 2024; Rahma et al., 2023).

Research on willingness to participate in medical research shows that trust in researchers (p < 0.001), prior exposure to research (p = 0.006), and the perceived invasiveness of a study, with low-invasiveness providing the highest likelihood with 79.1%, all influence participation decisions. This has direct implications for PGx research expansion because building representative genomic datasets requires broad and sustained community engagement. Transparent governance, careful consent practices, and visible public benefit will be essential if genomic initiatives are to maintain legitimacy and inclusiveness (Hajjar et al., 2025; Rahma et al., 2021a).

## 6 Healthcare professional readiness

Available studies consistently show that healthcare professionals generally hold favorable attitudes toward pharmacogenomics, yet their practical knowledge remains uneven. Surveys of healthcare workers, which included 552 respondents, found high levels of positive attitude toward genetic testing and PGx (91.9%), but only moderate theoretical understanding with a mean score of 5.2/9 (Rahma et al., 2020a). Similar patterns were observed among medical and health sciences students, indicating that support for the concept of precision medicine often exceeds confidence in its real-world application (Rahma et al., 2020b). This is a common early-stage implementation pattern and highlights a concrete opportunity for targeted workforce development (Jairoun et al., 2024; Ramadan et al., 2024).

Pharmacists occupy a particularly important position because they are often expected to serve as accessible medication experts within the healthcare system. However, UAE cross-sectional studies, conducted using qualitative methods, showed that 70% of the 399 pharmacists surveyed, especially in community settings, reported insufficient PGx training and felt limited in their ability to contribute meaningfully to testing decisions or therapeutic recommendations (Rahma et al., 2020c). High prescription volume and workload were also associated with weaker knowledge and less favorable attitudes (p = 0.001), suggesting that implementation barriers are not purely educational but also organizational. Without protected time, structured protocols, and formal scope of practice support, pharmacists may remain underused in PGx delivery despite their potential central role (Jairoun et al., 2024; Ramadan et al., 2024).

Encouragingly, interprofessional education initiatives have demonstrated that workshops involving 248 students from medicine (47.6%), pharmacy (36.7%), dentistry (13.3%), and health sciences (2.4%) can improve understanding of patient safety, precision medicine, and collaborative care (p < 0.001) (Guraya et al., 2025). These findings suggest that PGx training should not be confined to a single profession but instead embedded in multidisciplinary curricula and continuing professional development. Because PGx recommendations often sit at the intersection of prescribing, dispensing, counseling, and laboratory interpretation, interprofessional competency is particularly important for safe implementation (Guraya et al., 2025).

## 7 Educational and system-level challenges

The educational environment remains one of the clearest structural barriers to pharmacogenomics implementation. Mapping studies have shown that PGx is inconsistently integrated across the 21 accredited universities in the UAE, with only six universities offering formal PGx education. Even where included, the curriculum is often limited to one or two credit hours and is primarily confined to pharmacy programs (Rahma et al., 2021b). This fragmented exposure contributes to the pattern in which professionals and students are supportive of PGx in principle but insufficiently prepared to use it in practice. Curricular integration must move from isolated coverage to longitudinal training that spans undergraduate education, clinical internships, and postgraduate development (Rahma et al., 2021a).

This need is particularly important in healthcare systems that rely on a highly diverse workforce, where professional education must serve a highly diverse healthcare workforce with variable prior training and different cultural perspectives on genetics and privacy (Rahma et al., 2021c).

## 8 Strategic priorities for implementation

The UAE has established many of the foundational components required for successful pharmacogenomics implementation, including national genomic initiatives, population-specific genomic resources, and emerging evidence for clinically actionable pharmacogenetic variation (Abbas et al., 2025b). However, translating these advances into routine clinical practice will require a coordinated national strategy (Ateia et al., 2023; Rahma et al., 2023; Rahma et al., 2021a; Abbas et al., 2025b).

First, pharmacogenomics implementation should initially focus on therapeutic areas with the strongest local evidence base, particularly cardiovascular medicine, where variants affecting warfarin, clopidogrel, and statin therapy have already been identified in UAE populations. Pilot implementation programs in these settings could generate local clinical and economic outcome data to support broader adoption (Al-Mahayri et al., 2022; Abbas et al., 2025b).

Second, continued investment is needed to expand UAE-specific genomic reference resources and pharmacogenomic databases. Population-specific variant annotation, clinical curation, and decision-support tools will be essential to ensure that prescribing recommendations accurately reflect the genetic diversity of Emirati and UAE-resident populations (Ateia et al., 2023; Marzouka et al., 2024; Daw Elbait, 2021).

Third, integration of pharmacogenomic information into electronic health records and clinical decision-support systems should be prioritized to facilitate practical implementation at the point of care. Such systems can assist clinicians in interpreting genetic results and applying evidence-based prescribing recommendations (Al-Mahayri et al., 2022; Hajjar et al., 2025; Guraya et al., 2025; Rahma et al., 2021b; Abbas et al., 2025b).

Fourth, educational initiatives targeting healthcare professionals should move beyond awareness and focus on developing practical competencies in test interpretation, patient counseling, and pharmacogenomic-guided prescribing. Interprofessional training programs involving physicians, pharmacists, genetic counselors, and laboratory specialists may be particularly valuable (Rahma et al., 2021b).

Finally, sustainable implementation will require supportive regulatory frameworks, reimbursement policies, public engagement initiatives, and continued evaluation of clinical utility and cost-effectiveness. Collectively, these efforts will help establish a robust precision medicine ecosystem capable of supporting long-term pharmacogenomics integration throughout the UAE healthcare system (Rahma et al., 2021c; Abbas et al., 2025b).

## 9 Conclusion

This review highlights the substantial progress achieved in pharmacogenomics within the UAE. Studies of Emirati-based populations have identified genetic determinants of variability in warfarin dosing, clopidogrel response, statin-associated adverse effects, ACEI intolerance, HLA-related drug safety, and diabetes-related outcomes (Al-Mahayri et al., 2022; Abbas et al., 2025b), highlighting the potential of precision medicine to improve therapeutic decision-making.

Future progress will depend on translating genomic discoveries into routine healthcare through workforce development, clinical decision-support systems, local guideline development, and sustainable reimbursement models. With continued investment and coordinated national efforts, the UAE is well-positioned to become a regional leader in pharmacogenomics and precision medicine.
